# Salivary citrullinated proteins in rheumatoid arthritis and associated periodontal disease

**DOI:** 10.1038/s41598-021-93008-y

**Published:** 2021-06-29

**Authors:** Ildikó Tar, Éva Csősz, Edit Végh, Karin Lundberg, Nastya Kharlamova, Boglárka Soós, Zoltán Szekanecz, Ildikó Márton

**Affiliations:** 1 Department of Oral Medicine, Faculty of Dentistry, University of Debrecen, Debrecen, Hungary; 2 Centre for Molecular Medicine, Faculty of Medicine, University of Debrecen, Debrecen, Hungary; 3 Division of Rheumatology, Department of Medicine, Faculty of Medicine, University of Debrecen, Nagyerdei str 98, Debrecen, 4032 Hungary; 4 Division of Rheumatology, Department of Medicine Solna, Karolinska Institutet, Stockholm, Sweden; 5 Department of Restorative Dentistry and Endodontics, Faculty of Dentistry, University of Debrecen, Debrecen, Hungary

**Keywords:** Immunology, Rheumatology

## Abstract

Periodontal disease (PD) can be an important precipitating factor in the production of citrullinated proteins. Its importance is emphasized, but it is not the only way to produce citrullinated proteins. The aim of the current study was to determine the periodontal conditions and the salivary citrullinated protein content in patients with rheumatoid arthritis (RA) compared to healthy controls. We also wished to correlate citrullinated protein levels in the saliva and serum biomarkers with the periodontal status and temporomandibular joint (TMJ) involvement of patients with RA. Twenty-three patients with RA and 17 healthy controls participated the study. Saliva samples were taken: citrulline content of saliva was measured. Blood test results for patients with RA were collected. TMJ disorders were described. Cariological and periodontal indices were registered. Periodontal conditions and periodontal staging were also registered. Comparison of measured values between groups was performed. Intragroup correlation of patients’ values was counted. The prevalence of TMJ complaints was significantly higher in the RA group (8/23) versus controls (1/17). The patients with RA had worse periodontal condition because more patients with RA had gingivitis with a significantly higher bleeding on probing (BOP) (RA: 22.4 ± 25.0%; controls: 6.36 ± 11.6%; *p* = 0.018). Gingival index (GI) was also significantly higher in the patients than in controls (RA: 0.68 ± 0.58; controls: 0.19 ± 0.38; *p* = 0.010). The citrullinated protein (relative) content of saliva did not differ significantly (*p* = 0.147) between patients with RA (1102.2 ± 530.8) and healthy controls (1873.1 ± 1594.9). In RA, the salivary anti-CCP levels positively correlated with PD staging (R = 0.464, *p* = 0.039) . Control subjects more commonly had healthy gingiva than RA patients. Moreover, in the control group more individuals had intact and reduced height periodontium than periodontitis compared to the RA group. There was no significant difference in the levels of salivary citrulline between patients with RA and controls, despite the significant differences in their periodontal status. Thus, salivary citrulline levels are not associated with RA disease severity.

## Introduction

The amount of citrullinated proteins is higher in inflamed tissues. This is typical for rheumatoid arthritis (RA), where the synovial tissue expresses high amounts of citrullinated proteins^1,2^. This induces the formation of anti-citrullinated protein antibodies (ACPAs). There are different types of ACPAs that are used for the diagnosis of RA: anti-cyclic citrullinated peptide (anti-CCP), anti-mutated citrullinated vimentin (anti-MCV), anti-citrullinated fibrinogen (anti-CF), anti-citrullinated enolase peptide (anti-CEP) and others^1–5^. According to previous studies, *Porphyromonas gingivalis (P.gingivalis),*anti-CCP and anti-CEP can be detected in patients with both RA and periodontal disease (PD)^2,6,7^. Anti-CEP cross-reacts with bacterial enolase and has been associated with radiographic progression in RA^7,8^.

Certain anti-citrullinated peptides have been associated exclusively with the presence of *Prevotella intermedia (P.intermedia)*^9^. This bacterium is in close interaction with *P.gingivalis* in the dental plaque. *P.intermedia* also initiates interleukin 17 (IL-17) mediated inflammation^10^. König and Andrade^11^ demonstrated the role of *Aggregatibacter actinomycetemcommitans (A.actinomycetemcommitans)* in the dysregulation of peptydil arginine deiminases (PADs) in neutrophils by inducing hypercitrullination stimulated by leukotoxin A of this bacterium. *A. actinomycetemcommitans* is a primary periodontopathogen, a Gram negative, facultatively anaerobic short rod that forms homogenous layers and initiates the shift from acute homeostatic inflammation (initial lesion) to pathologic chronic inflammation (early lesion) in the gingiva^12^. *P.gingivalis*, which is a Gram negative, obligate anaerobic periodontopathogenic bacterium^13^, and connate bacteria augment the periodontal inflammation initiated by *A. actinomycetemcommitans*^11,12^.

Citrullination is a physiological process resulting in citrullinated proteins. The end product is citrulline that is more hydrophobic than arginine itself^1,2^. Under certain circumstances the citrullination process could be pathological, resulting in altered self-antigens, which may be converted to autoantigens^1,2^. One of the executors of non-physiological citrullination is the bacterial PAD (PPAD) enzyme of *P. gingivalis*. Its enzymatic activity is similar to human PADs, however, it can target proteins which are normally not accessible for human PADs^2,14^.

Ögrendik et al.^14^ suggested that the PPAD of *P. gingivalis* could be one of the contributors of converting self-antigen to autoantigen. The original role of bacterial enzymatic activity is to ensure proper nutrient supply from the surrounding tissues for the periodontopathogenic bacteria. This citrullination process also enables further periodontopathogenic bacterial colonization and multiplication^14^, such as *Prevotellae* and *Flavibacteria* species^15^.

PD may be an important precipitating factor in the production of citrullinated proteins^2^. Products of periodontal inflammation are released to the gingival crevicular fluid (GCF). This is an exudate flowing through periodontal crevice, sweeping out planktonic bacteria from the area^16^. Finally in the oral cavity this fluid is mixed with saliva^12^. The other source could be the citrullinated proteins of the serum. Yet, there is no data on the amount of citrullinated proteins in saliva in relationship with RA.

We have not yet found any publications on salivary citrulline in context with RA. The aim of the current study was to determine the periodontal conditions and the salivary citrullinated protein content in patients with RA compared to healthy controls. We also wished to correlate the levels of citrullinated proteins in the saliva and certain serum biomarkers (anti-CCP and anti-CEP with the periodontal status and temporomandibular joint (TMJ) involvement of patients with RA.

## Materials and methods

### Patients and controls

Twenty-three unselected patients with RA (22 females and one male) were recruited for the study. Diagnosis of RA was established according to the EULAR/ACR 2010 classification criteria by rheumatologists^17^. The mean age ± SD was 57.3 ± 11.0 (range: 23–74) years. The mean disease duration was 10.0 ± 3.8 (range: 2–27) years. The mean disease activity (DAS28) was 3.82 ± 0.25 (Table 1). Out of the 23 RA patients, 19 received methotrexate, 10 received biologics (no JAK inhibitors) and 7 were on low-dose corticosteroids at the time of recruitment to the study. Patients were referred by the Division of Rheumatology, University of Debrecen Faculty of Medicine to the Department of Periodontology, University of Debrecen Faculty of Dentistry. Age and gender matched controls (n = 17, 16 females and one male), were also recruited. Their mean age ± SD was 55.6 ± 6.9 (range: 47–66) years (Table 1). The study was performed according to the Declaration of Helsinki and had been approved by the Hungarian National Ethical Review Board (ETT-KFEB, No. 3386–2011). All patients and controls signed informed consents.

**Table 1:** Characteristics of RA patients and healthy controls.

	RA patients (n = 23)	Healthy controls (n = 17)
Age (mean ± SD)	57.3 ± 11.0	55.6 ± 6.9
Male gender (n)	2	1
Disease duration (mean ± SD)	10.0 ± 3.8	–
Disease activity (DAS28)	3.82 ± 0.25	–
RF seropositivity, n (%)	17 (74)	–
Anti-CCP seropositivity, n (%)	16 (70)	–
Anti-CEP positivity, n (%)	10 (43)	–
Allergy (n)	4	0
Smoking (n)	4	1
TMJ complaints* (n)	8	1
Dry mouth symptoms (n)	1	0
DMFT (mean ± SD)	19.87 ± 8.19	17.50 ± 8.83
DMFS (mean ± SD)	91.48 ± 49.78	74.58 ± 51.78
Edentolous (n)	3	0
MT* (mean ± SD)	13.48 ± 9.63	7.36 ± 6.17
BOP(%)* (mean ± SD)	22.4 ± 25.0	6.4 ± 11.6
GI* (mean ± SD)	0.8 ± 0.7	0.2 ± 0.5

BOP, bleeding on probing; CCP, cyclic citrullinated peptide; CEP, citrullinated enolase peptide; DAS28, 28-joint disease activity score; DMFT, sum of decayed + missing + filled teeth; DMFS, sum of decayed + missing + filled teeth surfaces; GI, gingival index; MT, number of missing teeth; RA, rheumatoid arthritis; RF, rheumatoid factor; SD, standard deviation; TMJ, temporomandibular joint.

*Significant difference between patients and controls, *p* < 0.05.

### Clinical assessment

TMJs were examined according to standard methods to reveal possible involvement both the patient and control groups^18,19^. Saliva samples were taken by the spitting method into a 50 mL centrifuge tube^20^. The amount of saliva was measured after 5 min by reading the tube’s scale. Saliva was examined as described later. After saliva collection, patients’ history was explored, and oral examination was performed by the very same study investigator (IT). Severity of PD and dental caries experience were assessed^21,22^.

Dental charting was performed for each patient. DMFT (sum of decayed, missing, and filled teeth) and MT (number of missing teeth) indices were determined^23^.

Characterization of periodontal disease included full-mouth measurements with the help of a periodontal probe: determination of plaque (PI) and gingival (GI) indices, bleeding on probing (BOP), and the distance of cemento-enamel junction—coronal marginal gingiva (GM-CEJ), clinical probing depth (CPD) and clinical attachment loss (CAL) were registered in mm. Attachment loss (AL) was counted as the sum of GM-CEJ and CPD. X-ray examination was also done. MT was counted. PD staging was decided based on findings according to Tonetti et al.^21^.

The diagnosis of PD was based on previous studies^24^ and according to classification criteria published by Lang and Bartold^25^ and Caton et al.^22^. Patients were classified as outcomes of periodontal health: pristine gingiva, clinically healthy gingiva, gingivitis, stable periodontal disease, controlled periodontal disease and uncontrolled periodontitis^22,25^.

### Sample collection and processing

Unstimulated saliva samples were collected from all 23 patients with RA and 17 control subjects. All individuals were asked to avoid eating, drinking or performing oral hygiene measures for at least one hour before the sample collection. They also had to rinse their mouths with water prior to saliva donation. Saliva samples were kept on ice throughout the collection and processing. No more than 60 min elapsed from the time of sample collection to freezing. Samples were centrifuged at 4100×*g* for 15 min at 4 °C and the supernatants were transferred to fresh tubes and kept on −70 °C until further processing.

### Protein concentration

All reagents used in this study were of at least analytical grade and were purchased from Sigma-Aldrich (St. Louis, USA) if not stated otherwise.

The protein concentration of saliva samples was determined by using Bradford reagent (Sigma-Aldrich, St. Louis, USA). The assessment was performed according to the manufacturer’s instructions.

### Peptidyl-citrulline level determination with immuno(dot)blot method

Our group has developed a method for the examination of citrullination^26^, which can be used for the identification of citrullinated proteins in biological samples. Saliva samples corresponding to 20 µg total salivary protein were used for dot-blot analysis. The PVDF (VWR International Ltd., Radnor, Pennsylvania, USA) membrane was activated by methanol and the samples were dropped onto the membrane in duplicates. Blocking was carried out using 1% polyvinylpirrolidone PVP-40 in TTBS buffer (10 mM Tris, 150 mM NaCl, pH 8.0) for 60 min at room temperature^27^. The membrane was incubated with 1:15,000 anti citrulline mouse polyclonal antibody (PAB0672, Covalab UK Ltd. Cambridge, UK) at 4 °C overnight, followed by incubation with 1:15,000 horse radish peroxidase (HRP)—conjugated anti-rabbit IgG antibody (Sigma-Aldrich, St. Louis, USA) for 1 h at room temperature. The membrane was visualized using ECL substrate detection kit (Thermo FisherScientific Inc, Waltham, MA, USA) and developed on autoradiographic film (Agfa, Motsel, Belgium). Densitometry was carried out by using QuantityOne 4.6.7 software (BioRad Ltd., Hercules, CA, USA) and individual background substraction was applied. Density values are presented relative to the average density of 5 parallel ferryl (IV)-hemoglobin reference spots.

### Laboratory tests

Serum levels of IgM-type rheumatoid factor (RF) and C-reactive protein (CRP) were assessed by quantitative nephelometry (Cobas Mira Plus, Roche), using RF and CRP reagents, respectively (both Dialab, Vienna, Austria). RF levels > 50 IU/mL and high sensitivity CRP levels > 5 mg/L were considered elevated. Anti-CCP autoantibodies were detected in serum samples using the Immunoscan-RA CCP2 ELISA test (Euro Diagnostica, Arnhem, The Netherlands). The assay was performed according to the instructions of the manufacturer. A concentration > 25 IU/ml was considered positive. All these tests were performed in the Department of Laboratory Medicine of the University as part of the follow-up protocol for patients with RA. The salivary anti-CCP autoantibodies were examined with Anti-CCP EDIA kit (Euro Diagnostica, Malmo, Sweden) according to the manufacturer’s instructions in a qualitative setup. Absorbance ratios were calculated according to the manufacturer’s recommendations and values > 1 were considered positive.

### Determination of anti-CEP-1 levels

Anti-CEP-1 IgG was measured in the serum samples using an in-house peptide ELISA, as previously described^28^. Anti-CEP-1 IgG levels are presented as arbitrary units/ml (AU/ml), based on a standard curve. The cut-off for positivity was > 3.7 AU/mL^28^. The test were performed at Karolinska Institutet.

### Statistical analysis

Statistical processing of data was performed by using the SPSS 22.0 software. Means, standard deviation, minimum and maximum of variables were calculated. Comparison of means were performed with t-test to decide significant differences between diseased and test groups variables. Intragroup evaluation of variables of the patients with RA were entered to Pearson’s correlation tests to reveal possible relationships of measured variables. P values < 0.05 were considered statistically significant.

### Ethics approval and consent to participate

The study was performed according to the Declaration of Helsinki and had been approved by the Hungarian National Ethical Review Board (ETT-KFEB, No. 3386–2011). All patients and controls signed informed consents. No individual patient data are presented.

### Consent for publication

Non applicable.

## Results

The main characteristics of patients with RA and controls are included in Table 1. In both groups, similar frequencies of individuals with allergy (RA: 4/23, healthy 0/17), smoking (RA: 4/23, healthy: 1/17), dry mouth symptoms (RA: 1/23, healthy: 0/17) were present (Table 1). There were no subjects with diabetes mellitus, pregnancy, or cancer in the study.

Cariological indices did not differ significantly between groups (DMFT *p* = 0.42, DMFS p = 0.36). DMFT of RA patients was 19.87 ± 8.19 (mean ± SD), while DMFT of healthy controls was 17.5 ± 8.83 (mean ± SD). DMFS of RA patients was 91.48 ± 49.78 (mean ± SD). DMFS of healthy controls was 74.58 ± 14.95 (mean ± SD). The only difference (*p* = 0.024) existed between groups in MT values with 13.48 ± 9.63 (mean ± SD) of patients with RA and 7.36 ± 6.17 (mean ± SD) for healthy controls. In the RA group, there were 3 edentolous (= no teeth) patients, while among controls there was none.

More patients with RA had gingivitis with a significantly higher mean BOP% (RA: 22.4 ± 25.0; controls: 6.36 ± 11.6; *p* = 0.018) (Table 1), and also GI was also significantly higher in patients with RA than in controls (RA: 0.68 ± 0.58; controls: 0.19 ± 0.38; *p* = 0.010).

According to the current classification of PD, in our patients with RA, two had intact periodontium (no AL). One of them had healthy gingiva (BOP˂10%) and the other had gingivitis (Table 2). There were 5 patients with RA who had reduced height periodontium. All these patients had healthy gingiva (Table 2). Out of the 13 patients with RA with periodontitis, 3 had healthy gingiva in PD stages 1 (n = 2) and 2 (n = 1). The other 10 patients with both RA and periodontitis had gingivitis with PD stages 1 (n = 4), 2 (n = 2), 3 (n = 3) and 4 (n = 1) (Table 3). In healthy controls, 6 had intact periodontium with BOP˂10%. Eight patients had reduced height periodontium (7 had BOP˂10% with 4 patients’ stage 1, and 3 patients with stage 2, 1 had stage 3 with BOP ≥ 10%). Control patients with periodontitis: 1 had stage 2 with BOP˂10%, while that 2 patients with BOP ≥ 10% had stage 3. According to current classification of periodontal diseases in PD staging (RA patients mean ± SD = 1.72 ± 0.95; healthy subjects’ mean ± SD = 1.77 ± 0.83, there was no significant difference between groups (*p* = 0.88) (Tables 2, 3).

**Table 2:** Periodontal status of RA patients and healthy controls.

	Healthy gingiva (x/n)	gingivitis (x/n)
**Intact periodontium**		
RA patients	1/23	1/23
Healthy controls	6/17	0/17
**Reduced height periodontium**		
RA patients	5/23	0/23
healthy controls	7/17	1/17
**Stable periodontitis**		
RA patients	3/23	10/23
healthy controls	1/17	2/17

RA: rheumatoid arthritis, healthy gingiva if BOP˂10%, gingivitis if BOP ≥ 10%. Note: three RA patients had no teeth at all.

**Table 3:** Classification matrix for staging in reduced height periodontium, and periodontitis patients.

	Stage	Reduced height periodontium (n)	Periodontitis (n)
RA patients	1	4	6
	2	1	3
	3	0	3
	4	0	1
Controls	1	4	0
	2	3	1
	3	1	2
	4	0	0

The prevalence of TMJ complaints were significantly higher in the RA group (8/23) vs controls (1/17) (*p* = 0.006). Symptomatic TMJ arthritis was associated with RA flares in other joints (Table 1).

Relative citrullinated protein content of saliva did not differ significantly (*p* = 0.147) between patients with RA (1102.2 ± 530.8) and healthy controls (1873.1 ± 1594.9). Serum anti-CCP levels for patients with RA mean ± SD was 269.82 ± 686.678 IU/mL (min–max: 5–3200), while salivary anti-CCP level mean ± SD was 1.59 ± 2.01 IU/mL (min–max: 0.273–7.38).

In patients with RA salivary anti-CCP levels positively correlated with PD staging (R = 0.464, *p* = 0.039) (Table 4). With respect to clinical examinations, there were significant correlations between variables, which relationships have already been scientifically established, as GI vs BOP (R = 0.655, p = 0.002), GI vs MT (R = 0.615, p = 0.005) and PI vs BOP (R = 0.627, *p* = 0.004) (Table 4).

**Table 4:** Significant correlations between variables in RA patients.

	Staging	BOP	MT
Serum anti-CCP	R = 0.464 *p* = 0.039	NS	NS
GI	NS	R = 0.655 *p* = 0.002	R = 0.615 *p* = 0.005
PI	NS	R = 0.627 *p* = 0.004	NS
TMJ	R = 0.591 *p* = 0.01	NS	NS

R: Pearson’s correlation coefficient. Abbreviations: BOP, bleeding on probing; CCP, cyclic citrullinated peptide,; GI, gingival index; MT, number of missing teeth; PI, plaque index;TMJ, temporomandibular joint. NS: not significant.

TMJ involvement exhibited positive correlation with periodontal disease staging (R = 0.591, *p* = 0.01) (Table 4.). Salivary anti-CCP levels of patients with RA seem to be independent from serum anti-CCP, RF, and salivary citrullinated protein levels, as there were no correlations among these parameters.

With respect to serum anti-CEP-1 assessments, 43% (10/23) of patients with RA were positive for anti-CEP-1 (levels > 3.7 AU/ml). While 100% of anti-CEP-1 positive patients were also IgM RF positive and anti-CCP positive, only 70% (9/13) of anti-CEP-1 negative patients were IgM RF positive and only 50% of them were anti-CCP positive. Anti-CEP-1 positive patients had significantly higher absolute anti-CCP levels (1252 ± 346 U/ml) compared to anti-CEP-1 negative patients (514 ± 182 U/ml; *p* < 0.05). The periodontal condition of anti-CEP positive and negative patients did not differ in a significant manner in terms of BOP (*p* = 0.77), but was different in terms of GI (*p* = 0.01). Their PD staging was the same (*p* = 0.86).

## Discussion

In our RA cohort, the patients more often had periodontal pathology compared to healthy controls. In addition, anti-CCP levels correlated with periodontal disease staging, while anti-CEP positive patients had more prominent gingivitis. Salivary citrullinated protein levels were similar in RA and controls.

The study group of patients with RA exhibited about the same smoker prevalence (17.3%) as in other studies (13.2%). Diabetic, pregnant, cancer patients were excluded from this study. Probably because of this, dry mouth symptoms were less frequent in our patients group (4.3%) than what was found in the COMORA study (18.6%)^29^.

In this study, patients with RA had similar DMFT and DMFS index values as healthy controls. The only difference between groups existed in MT which was higher in patients with RA. The prevalence of edentulousness was also higher among patients with RA. In accordance with an earlier study there was no difference between DMFT values of patients with RA and controls^30^. The significantly higher MT value of patients with RA compared to controls could also be seen in a previous study, but that study lacks the number of edentulous patients^31^.

In terms of periodontal condition GI, BOP, and the number of periodontitis patients proved to be significantly higher in the patient group. The staging of periodontitis cases assessed with the help of the new classification system did not show significant differences between patients with RA and healthy subjects^21,22^. In the control group there were more individuals with intact or reduced height periodontium. Despite the differences in periodontal condition (increased BOP, GI, more periodontitis cases, and higher MT) between groups, the citrullinated protein content of saliva was not significantly different. The citrullinated proteins of saliva might originate from gingival crevicular fluid and/or added during the active transport when saliva is produced in the salivary glands^32^. The difference in the prevalence of periodontal diseases in the two groups makes it more probable that the source of salivary citrullinated proteins must be the salivary gland^32^. They could be the result of physiological citrullination.

Salivary anti-CCP had shown significant correlation with periodontal staging in patients with RA. That supports the fact that salivary anti-CCP autoantibodies are produced in the periodontal tissues rather than in salivary glands^17,20^.

Control subjects more commonly had healthy gingiva than patients with RA. Moreover, in the control group more individuals had intact and reduced height periodontium than periodontitis compared to the RA group. This is in accordance with other reports^25,26^. However, reliable comparison between studies cannot be performed because of the different diagnosis protocols^20,27^.

Serum anti-CEP-1 positive patients with RA had significantly worse GI, but did not differ in periodontal staging at all from anti-CEP-1 negative patients. In a Korean study, anti-CEP-1 correlated with BOP and GI^29^. In our study, only GI was significantly higher in anti-CEP positive patients compared to anti-CEP 1 negative subjects.

We could just find a relationship between TMJ involvement and periodontal disease staging, which is somehow an obvious finding, as periodontal staging is based on MT, what is a risk factor for TMJ problems. Earlier there was a study, where significant relationship between serum anti-CCP and TMJ involvement was found^18^. However, we did not find any data regarding the possible relationship between salivary citrulline/ACPA and TMJ involvement. This finding is in accordance with reports previously published^18,19,33^.

There was no significant difference in the levels of salivary citrullinated proteins between patients and healthy controls, despite the significant differences in their periodontal status. Earlier, other authors observed that the amount of salivary TNF-α or anti-*Porphyromonas* IgG was also not proportionate with periodontal inflammation. The same was reported on serum anti-CCP antibodies^34^. The production of citrullinated proteins could be the result of the daily regeneration of oral tissues, and/or the activity of PPAD producing bacteria in the gingival crevice who releases their products to the saliva.

The parallel evaluation of blood test results and periodontal chart values conclusively led to the significant relationships between serum anti-CCP and periodontal staging, however, CRP and salivary citrullinated protein levels did not show any correlations with periodontal status. With respect to serum anti-CCP, others did not find any significant relationships with periodontal condition ^34^.

Normally, physiological citrullination will not exert ACPA production^35^. Pathological citrullination of protein arginine end chain may lead to the production of ACPAs. Pathological citrullination is proposed to be performed in several ways. On one hand, there may be an increase in the amount of citrullinated proteins (hypercitrullination)^36^. On the other hand, there may be a quality change in citrullinated proteins, which are normally not citrullinated^1,2,14^. It is the *A. actinomycetemcommitans* periodontopathogenic bacterium, which secrets pore forming leukotoxin that may trigger endogenous PAD activity of neutrophils^11,12,37^. The other source of citrullinated proteins in the oral cavity is *P.gingivalis*. Another recently discovered oral bacterium that can also exhibit PPAD activities is *Mycoplasma argininii*^38^. The formation of certain ACPAs, such as anti-CEP, is also devoted to this pathogen. The bacteria having PPAD activity can transform locally expressed proteins into citrullinated proteins^2,6,7^. *P. intermedia* also a periodontopathogenic species also related to the citrullination of certain proteins^10^. All three bacteria are prominent periodontopathogenic microbes^39^. *A*.*actinomycetemcommitans* is the initiator of PD, while the other two may promote the progression of tissue destruction into periodontitis^2,7,10–12,28,37^.

In individuals at high risk for RA the IgA isotype of ACPA is predominant suggesting the involvement of mucosal surfaces in the process of autoantibody formation^2,40^, though there are opposite findings as well^41^. In this study, we could not assess IgA ACPA. The other common pathogenic pathway could be polarized T_H_ cell differentiation and the activation of T_H_17 cells. This process also occurs in PD^42^.

The strengths of our study is the original idea of measuring citrullinated proteins in the saliva of RA patients. Limitations of the study: the number of patients available for the study. Patients had been selected and referred by the rheumatologist in charge for the patients. At the time of the examination GCF sample collection was not considered. The results of saliva measurements would have indicated further analysis of GCF, but at that point the patients were put on biological therapy. Perhaps, that would have changed the possible results.

We are also aware that not having qualitative data about the citrullinated proteins is a limitation of our study. We have tried several methods for the examination of protein citrullination. We developed a well-performing, reliable method, dot blot^26^. The ultimate method for identification of citrullinated proteins would be mass spectrometry. In the future we would like to continue our project with the examination of saliva samples with mass spectrometry and with the identification of citrullinated proteins in RA and other pathological conditions.

## Conclusions

In conclusion, in our study we confirmed that PD is more common in patients with RA, but its pattern is not different from controls. Anti-CCP antibody production correlated with the stage of PD. Moreover, anti-CEP-1 positivity predicts a higher level of inflammation in the gingiva. Our study suggests, the PD is important in the pathogenesis of RA, however the major characteristics of RA-related and primary PD do not show major differences. Salivary citrulline content alone does not seem to be a good marker for the prediction of RA.

## Data Availability

Data can be obtained from the authors upon request.
